# The Relationship between Demographic Variables and Diabetes Self-Management in Diabetic Patients in Amman City/Jordan

**DOI:** 10.5539/gjhs.v5n2p213

**Published:** 2012-01-24

**Authors:** Mezyed A. Adwan, Yahya W. Najjar

**Affiliations:** 1 Zarqa University College, Al-Balqa’a Applied University, Zarqa, Jordan

**Keywords:** diabetes mellitus, diabetes self-management, demographic variables, income

## Abstract

**Background:**

Diabetes is a chronic disease that requires routine and complicated self care. Although self care can be managed by most diabetes patients, there are many variables that may make diabetes self-management difficult.

**Aim:**

The study examined the relationship between clients’ demographic variables and diabetes self-management in diabetic clients in Amman city/Jordan.

**Method:**

The data were collected through a self-completed questionnaire developed by the researchers and combined with the perceived diabetes self-management scale (PDSMS). The sampling of the investigation comprised 178 diabetes clients from Amman city/Jordan.

**Findings:**

There was proportional little relationship between income level and diabetes self management, and reversely proportional low relationship between duration of diabetes and diabetes self management. Other variables had no relationship with diabetes self management.

**Conclusion:**

The demographic variables related to diabetes self-management in this study are income level and duration of diabetes. As income level increases, diabetes self-management become better, and the longer the duration of diabetes, the worse is diabetes self-management.

## 1. Introduction

Diabetes mellitus (DM) is a devastating chronic disease (Donnell et al, 2001). About 30% of Arab people with diabetes, and 624 million Jordanian Dinars (JD) are spent on diabetes treatment every year (Al- Ajlouni, 2010). More than 70% of diabetic persons die of macrovascular disease associated with myocardial infarction and stroke (Donnell, Hoehns, Sutherland, & Wiblin, 2001). With the gradual lengthening of the human life expectancy and an increasingly sedentary lifestyle, the number of people with diabetes has been steadily increasing (Chen, Huang, & Yeh, 2009). Medical care in the absence of adequate self-care is rarely effective for chronic illnesses (Bonsignore & Suhl, 2006), and DM is a complex disease to manage, and most of the care involving self-management (Chlebowy, Hood & LaJoie, 2010), so people with diabetes must be equipped with the information necessary to implement the routine and complicated adequate self-care (Chen et al., 2009), these information include educating about home blood glucose monitoring, meal planning, and recognizing how and when to administer insulin or take oral diabetes medications (Bonsignore & Suhl, 2006).

Many variables may affect diabetes self-management. According to the health belief model (Rosenstock, 1974), it is postulated that individual perception of susceptibility and seriousness of a disease and perceiving the benefits of and barriers to disease screening tests and therapeutic regimen are ascribed to demographic, psychosocial and structural factors. In addition, external factors facilitates adherence to diabetes self-management which include support from family, peers, and health care providers behaviors by providing cues to action, direct assistance, reinforcement, and knowledge about diabetes. However, internal factors may be barriers to diabetes self-management which include fears associated with glucose monitoring, lack of self-control over dietary habits, memory failure, and perceived lack of personal control over diabetes (Chlebowy, Hood, & LaJoie, 2010).

Self-efficacy is another variable that has an impact on diabetes self-management behaviors regarding choosing and adhering to optimal diet, regular exercise, self monitoring of blood glucose and foot care (Fisher, Sarkar, & Schillinger, 2006). Patients with diabetes and low literacy have poor knowledge of their disease and may have difficulties learning the advanced self-care skills needed to improve glycemic control. However, focusing on selected critical behaviors, decreasing the complexity of information, using concrete examples, limiting the number of topics covered in one session, avoiding jargon, and using “teach back” to ensure comprehension were effective strategies for communicating with patients with low literacy, therefore improving self care and outcomes (Bryant et al., 2004). Individualizing diabetes self management education based on client's age, cultural influences, health beliefs and attitudes, diabetes knowledge, self-management skills and behaviors, readiness to learn, health literacy level, physical limitations, family support, and financial status may be more effective for diabetes self management (Brown et al., 2009). Charlotte (2007) recommended techniques to improve communication between health care providers and patients with inadequate literacy, these include making more frequent use of oral and visual instructions; limiting instructions to essential information only; making instructions interactive, with patients demonstrating their understanding of the topic; and encouraging the assistance of surrogate readers, who accompany patients during clinic visits to assist them in navigating the health care system.

Bush et al (2004) reported that depressed patients adhered less to oral hypoglycemic agents and antihypertensive and lipid-lowering medications, so diabetic patients with depression need support for self-management activities such as lifestyle modifications and medication adherence. Type 1 diabetes males have low adherence to diabetes regimen and treatments compared with Type 1 diabetes females (Cakan et al., 2006). Many people with diabetes experience problems due to medication costs, and asking patients about their ability to afford treatment is important for diabetes control and self management (Brown et al., 2009)

Time pressure may be another factor affecting diabetes self-management. According to Russell, Safford and Suh (2005), elderly patients and patients with long term complications of diabetes (neurological disorders/stroke, neuropathy, visual impairments, or depression) could require twice as long for most diabetes self-management tasks and might also need the help of a caregiver. They might not be able to carry out some tasks, such as exercise, because of lack of sufficient time, especially with presence of physical limitations.

A survey conducted by Ajlouni et al. (2008) in Sarih town in Irbid city, Jordan has revealed that DM in diagnosed clients and receiving treatment was not controlled, which indicates that treatment was not effective or there is a lack of compliance with the treatment protocol. It is possible that patients rely much on medications and ignore the need to modify their eating habits and increase their physical activity. The survey has also revealed that DM was significantly related to age, body mass index, and family history of diabetes and was high among those with the low education level.

## 2. Purpose of Study

This study aimed to examine the relationship between demographic variables (age, gender, income, marital status, education level, profession, duration of diabetes, DM type) and diabetes self-management in diabetic clients in Amman City/Jordan.

## 3. Method

### 3.1 Instrumentation

The data were collected using a self –completed questionnaire (survey). An author-developed profile (items created by the author not present originally in the instrument) was used to obtain demographic and personal information (i.e.: age, gender, marital status, income, etc…), in addition to the Perceived Diabetes Self-Management Scale (PDSMS) (Cherrington, Rothman, & Wallston, 2007). PDSMS is 8-item self-report questionnaire measuring the self-motivation, attitude and capacity of client to adhere to therapeutic regimen of DM (Cronbach's alpha for the eight items was 0.83). The responses for the PDSMS items range from 1= ‘strongly disagree’ to 5= ‘strongly agree’ (Table 1). The questionnaire was anonymous (had a cover page included the study topic) and none of the participants knew it nor saw it before nor completed or being asked the questions in it before (it has not been used in Amman before).

**Table 1:** Perceived diabetes self-management scale (PDSMS)

Items	Score				

	Strongly disagree	Disagree	Uncertain	Agree	Strongly Agree
1. It is difficult for me to find effective solutions for problems that occur with managing my diabetes.*					
2. I find efforts to change things (problems and bad health habits) I don’t like about my diabetes are ineffective. *					
3. I handle (manage) myself well with respect to my diabetes.					
4. I am able to manage things (problems or regimens) related to my diabetes as well as most other people.					
5. I succeed in the projects I undertake to manage my diabetes.					
6. Typically, my plans for managing my diabetes don’t work out well.*					
7. No matter how hard I try, managing my diabetes doesn’t turn out the way I would like.*					
8. I’m generally able to accomplish my goals with respect to managing my diabetes.					

* Item needing to be reverse-scored [(1 = 5), (2 = 4), (4 = 2), (5 = 1)]

### 3.2 Sampling

The study used a convenient sample from the clients with diabetes who live in Amman City (N = 902). Half of study population were males, and the other half were females. About 40% or more of the study population are 30 years old and older. According to G-Power 3.0.3 (Faul & Erdfelder, 1992) with a medium effect size of 0.3 and power of 0.80 and at 0.05 two-tailed level of significance, the required sample size will be at least 178 clients. The sample selected depending on assuring that diabetic clients are present in the health care center, in 3 medical health centers (Abu-Nsair medical health center, Amman comprehensive medical health center, and Al-Hashmi Al-Shamali medical health center) and within the period from may/2011 to August /2011 there were six health education sessions about diabetic foot care and prevention, and the participants were selected after finishing the sessions.

Participants were selected based on the following:

1) age 18 years or older: (in order to understand and answer the self-reported questionnaire clearly not based on chance).
2) diagnosed with diabetes.
3) received health services in the centre for at least six months.
4) lives in Amman city and its municipalities.

Clients excluded from participating in the study were as follows: age under 18 years, and who lives outside Amman city.

### 3.3 Data Collection

180 clients were chosen according to the selection criteria mentioned above (about 30 in each health education session). The selected clients were given the self – completed questionnaire during the visit to the medical centres for attending the health education session and were asked to complete the questionnaire within 15 minutes and submitting it immediately after leaving the session, or participants with low literacy level were interviewed for 15 minutes by the author asking the questions in the questionnaire. The self – completed questionnaire had a cover letter that includes information about the purpose of the study and what is expected from them and where to return the questionnaires, and that the study is anonymous. In addition, the cover letter included contact information of the principal investigator and co-investigator for any further information and for answering the questions related to the study.

Participants who had low literacy completed the questionnaire by being interviewed by the authors and asking them the questions included in the questionnaire within 15 minutes. Participants’ family members accompanying them were asked to complete the questionnaire (writing the answers) after being answered verbally by the participants.

Permission to enter the health education sessions was gained by acknowledging the head persons of the medical health care centers about the study aims and target population. The cover letter of the questionnaire and the questions within it contained no identification numbers about participants and they were not asked to write their names unless they want to do. In addition, they signed the cover letter that guaranteed the confidentiality of their data.

### 3.4 Statistical Analysis

All missing data were treated using the method of mean replacement (for variables with normal distribution, or median replacement for variables with skewed distribution).

Pearson correlation Coefficient (Pearson r) were used to test the correlation between demographic variables and diabetes self-management. Descriptive statistics were used to describe frequencies of variables, and the answers for eight items of PDSMS were summated then correlated to each demographic variable.

Statistical analyses performed using computer program SPSS (Statistical Package for the Social Sciences).

## 4. Results

Response rate of participants were 98.9% (questionnaire distributed and submitted at the same time during the visit to medical health center. Only 2 participants did not return the questionnaire)

Table 2 shows the frequencies and percentages of categories for each demographic variable. More than half of the participants were with low annual income (less than 2000 JD) and more than eighty percent of the participants depend on the insurance coverage for medical treatment and regular follow-up. Less than half of the participants had duration of diabetes of more than 5 years.

**Table 2:** Frequencies and percentages of demographic variables (n = 178)

Variable	Categories	Frequencies	%	Variable	Categories	Frequencies	%
Gender	Male	50	28.1	Education level	Illiterate	9	5
	Female	128	71.9		Primary	54	30.3
Age	20 – 40	9	5		Secondary	53	29.8
	41 – 60	112	62.9		Diploma	29	16.3
	61 – 80	56	31.5		Baccalaureate	25	14
	More than 80	1	0.5		Master	6	3.4
Annual income	Less than 1000 JD	78	43.8		Doctorate	2	1.2
	1001 – 2000	33	18.5	Marital status	Married	141	79.2
	2001 – 3000	23	13		Single	8	4.5
	3001 – 4000	22	12. 35		Widow	26	14.6
	More than 4000	22	12.35		Divorced	3	1.7
Job	Private sector	19	10.7	Insurance coverage	Ministry of health	82	46.1
	Public sector	18	10.1		Military forces	29	16.3
	Not working	108	60.7		University	12	6.7
	Retired (civil)	24	13.5		Private sector	20	11.2
	Retired (military)	9	5		Cash payer	24	13.5
Duration of diabetes	Less than 1 year	47	26.4		Royal bureau	11	6.2
	1 – 5 years	47	26.4	Diabetes type	Type 1	29	16.3
	More than 5 years	84	47.2		Type 2	124	69.7
						Other types	25	14

Table 3 shows the responses of participants to items of the PDSMS. Responses to items 1, 2, 6, and 7 were reverse- scored (i.e.: 5 = strongly disagree and 1 = strongly agree) because they represent states of bad self-management of diabetes. When comparing the responses for these items to each other, the responses were approximately the same. However, the responses to other items (3, 4, 5, and 8) were incongruent to the responses of items 1, 2, 6, and 7, which made it difficult to conclude that most participants had bad self-management of diabetes.

**Table 3:** Participants’ responses to Items of PDSMS (n = 178)

Item	Score	Frequencies	Percentages	Item	Score	Frequencies	Percentages
1	Strongly disagree	14	7.9	5	Strongly disagree	12	6.7
	Disagree	42	23.6		Disagree	11	6.2
	Uncertain	18	10.1		Uncertain	17	9.6
	Agree	72	40.4		Agree	102	57.3
	Strongly Agree	32	18.0		Strongly Agree	36	20.2
2	Strongly disagree	10	5.6	6	Strongly disagree	13	7.3
	Disagree	44	24.7		Disagree	50	28.1
	Uncertain	26	14.6		Uncertain	36	20.2
	Agree	62	34.8		Agree	48	27
	Strongly Agree	36	20.2		Strongly Agree	31	17.4
3	Strongly disagree	10	5.6	7	Strongly disagree	21	11.8
	Disagree	18	10.1		Disagree	46	25.8
	Uncertain	23	12.9		Uncertain	24	13.5
	Agree	90	50.6		Agree	48	27
	Strongly Agree	37	20.8		Strongly Agree	39	21.9
4	Strongly disagree	6	3.4	8	Strongly disagree	6	3.4
	Disagree	17	9.6		Disagree	25	14
	Uncertain	23	12.9		Uncertain	20	11.2
	Agree	100	56.2		Agree	87	48.9
	Strongly Agree	32	18.0		Strongly Agree	40	22.5

There was a significant correlation between annual income and PDSMS (p<0.05) and between duration of diabetes and PDSMS (p<0.01). Other variables had no significant correlation with PDSMS. Annual income and PDSMS were positively correlated with little relationship (Pearson r: 0.211). Duration of diabetes and PDSMS were negatively correlated with a low relationship (Pearson r: -0.256). Other variables were either positively or negatively correlated but with no significant correlation.

## 5. Discussion

The relationship between annual income and diabetes self-management is little (weak) and proportional, which means that as the annual income increases, the self-management of diabetes is controlled better. The strength of relationship is weak because most participants have low annual income. When the client is more able to afford new techniques and methods of diabetes self-management, diabetes will be controlled better. Furthermore, lack of self-control over dietary habits and appropriate meal planning may be managed by high income level (i.e. high income level can solve the problem of purchasing new species of food not included before in diet when diabetic clients plan for appropriate small and frequent meals recommended by dietitian). Furthermore, medication costs may be another problem experienced by diabetic patients (Brown et al., 2009).

The relationship between duration of diabetes and diabetes self-management is low and reversely proportional, which means that as the duration of diabetes increases, diabetes self-management and control decrease and thus become less effective. The relationship was low because less than half of the participants had duration of diabetes of more than five years. Long duration of diabetes may impair diabetes self-management, so support from family, peers, and health care providers behaviors by providing cues to action, direct assistance, reinforcement, and knowledge about diabetes (Chlebowy, Hood, & LaJoie, 2010) may be beneficial for diabetic clients and overcome the effect of duration of diabetes on self-management, especially when the long term complications (e.g.: retinopathy, neuropathy) begins to develop and possibly low adherence level developed by the long duration of the chronic disease. Perhaps time needed for diabetes self management may be prolonged for elderly patients and patients with long term complications of diabetes, and subsequent low adherence to diabetes self management and the need for the help of a caregiver (Russell et al., 2005)

Participants’ responses to items of PDSMS might be affected by the negative form of some items (items 1, 2, 6, and 7), which made incongruence among responses (i.e.: most participants had bad and good self-management at the same time), the incongruence made could be a reason of the low and weak relationships found between diabetes self-management and both income level and duration of diabetes. Re-arranging the items reflecting bad self-management successively (putting them the first four questions or the last four questions) may help the participants respond in more appropriate manner to these items.

Other demographic variables have no relationship with diabetes self management, so when dealing with diabetes clients to enhance self-management, income level and duration of diabetes should be taken in consideration because they may render diabetes control and self-management. Further research is recommended to detect the relationship between other demographic variables (age, gender, marital status, education level, diabetes type, insurance coverage, job) and diabetes self-management in clients who have high income level and long duration of diabetes to detect if there is any relationship between diabetes self-management and other variables.

## 6. Limitations of the Study

The results of this study are limited on diabetes clients in Amman city, so they cannot be generalized on other cities in Jordan. In Addition, the sample was convenient and is not representative for the study population. Understanding the language of the self-completed questionnaire and the PDSMS and choosing the best answer for each item (e.g. strongly agree or agree) might be another limitation to be addressed. In addition, the negative form used in the items of PDSMS might affect the participants’ responses (i.e.: does it reflect bad or good diabetes self-management). The authors recommend conducting similar studies on other cities in Jordan because Amman city are almost rural and have few or no villages or towns (most population have rural behaviors or characteristics).

## 7. Study Implications

The authors recommend the following:

- Direct assistance from family members or primary care providers is necessary for adherence to self-management of DM when the duration of DM exceed five years due to possible low adherence level by the long duration.
- Income level should be addressed when conducting health education about preventive measures of long term complications of diabetes and new self-management methods.
- Level of education, marital status, gender, diabetes type and job of client should not be considered as barriers to diabetes self-management when discussing and planning with the client for his/her chronic disease.

## 8. Conclusion

In conclusion, the results obtained indicated that there was a relationship between annual income and diabetes self-management (as income increases, the better diabetes self-management), and a reverse relationship between duration of diabetes and diabetes self-management (the longer the duration, the worse the diabetes self- management). Other variables have no significant relationship with diabetes self management. Emphasizing on assessing income level and duration of diabetes prior to discussing and planning diabetes self management for diabetic client is important for effective self management of diabetes, because time needed for self management skills and activities, degree of dependence and assistance of others and costs of equipment and materials may be a big deal for most diabetic clients.

## Appendix

Author developed profile for demographic data:

1. Gender: ( ) male ( ) female

2. Age: ( ) 20 – 40 years ( ) 41 – 60 years ( ) 61 – 80 years ( ) more than 80 years.

3. Annual Income in JD:

( ) less than 1000 JD ( ) 1001 – 2000 JD ( ) 2001- 3000 JD ( ) 3001 – 4000 JD ( ) more than 4000 JD

4. Profession (job area):

( ) private sector ( ) public sector ( ) not working ( ) retired (civil service) ( ) retired (military service)

5. Educational level:

( ) illiterate ( ) elementary ( ) secondary ( ) diploma degree ( ) baccalaureate degree ( ) Master degree ( ) PhD degree

6. Marital status: ( ) married ( ) single ( ) widow ( ) divorced

7. Insurance coverage institution:

( ) ministry of health ( ) armed forces ( ) university ( ) private sector

( ) cash paying ( ) coverage of the royal bureau

8. The duration of being a D.M patient:

( ) less than 1 year ( ) 1 – 5 years ( ) more than 5 years

9. The type of diabetes you are suffering from:

( ) Insulin Dependent Diabetes Mellitus (juvenile diabetes)

( ) Non- Insulin Dependent Diabetes Mellitus (adult diabetes)
